# H5N1 preparedness must integrate rural and agricultural realities

**DOI:** 10.1016/j.lana.2025.101186

**Published:** 2025-07-12

**Authors:** Bradley A. Firchow

**Affiliations:** Rural Physician Leadership Program, University of Kentucky College of Medicine, Morehead, KY, USA

Bartlett et al. provide a timely framework for responding to the growing threat of highly pathogenic avian influenza (H5N1).^1^ Their recommendations, including enhanced surveillance, decentralized testing, improved biosecurity, and vaccine readiness, reflect important lessons from past pandemics. However, successful implementation requires a more explicit focus: the social and infrastructural realities of rural agricultural communities where H5N1 risk is currently concentrated.

The H5N1 panzootic has largely unfolded in rural regions. Globally, these areas are marked by limited access to care, under-resourced public health infrastructure, and longstanding mistrust of institutions. Many agricultural workers, particularly in dairy and poultry production, face overlapping risks.^2^ These include occupational exposure, low wages, minimal health coverage, and few protections for reporting illness or seeking treatment. Without structural support such as guaranteed sick leave, accessible testing, and culturally attuned communication, many individuals at highest risk may be unable or unwilling to participate in containment efforts.

This concern is not hypothetical. Experiences during the COVID-19 pandemic revealed the failures of uniform messaging and top-down strategies in rural contexts.^3^ In contrast, trusted local actors such as cooperative extension agents, community health workers, and agricultural networks proved essential to improving vaccine uptake and public health engagement. H5N1 response strategies should build on this work by ensuring that efforts are developed in collaboration with rural stakeholders.

Regional variability in surveillance enforcement presents an additional challenge to national coordination. For example, USDA-mandated milk testing remains uneven across states, which limits the ability to monitor the spread of the virus in real time.^4^ Similarly, rural public health departments may lack the staffing and local broadband infrastructure needed to communicate test results, distribute guidance, or track outbreak data effectively. Federal implementation strategies must account for regional disparities in public health capacity.

Labor dynamics in the agricultural sector also require focused attention. Many agricultural workers are immigrants or seasonal laborers with limited legal protections and fear of retaliation.^5^ Without safeguards for anonymity and reporting, surveillance may miss early cases. Effective response requires language access, legal protections, and clear safety protocols.

The biological risk of H5N1 mutation warrants urgent action. However, technical solutions will not succeed unless designed to function within the realities of rural life. Surveillance efforts must complement community trust-building. Biosecurity guidance must be feasible in resource-limited settings. Communications strategies must be delivered through trusted messengers.^3^

Protecting public health will require systems that reflect the needs, limitations, and strengths of the communities most exposed.

## Declaration of interests

The author declare no competing interests.
